# Unreliable usage of a single influenza virus IgM antibody assay in influenza-like illness: A retrospective study of the 2016–2018 flu epidemic

**DOI:** 10.1371/journal.pone.0215514

**Published:** 2019-04-22

**Authors:** Yao Yao, Zhao Zhipeng, Song Wenqi, Li Runqing, Zhu Dong, Qin Kun, Zhao Xiuying

**Affiliations:** 1 Department of Clinical Laboratory, Beijing Children’s Hospital, Capital Medical University, National Center for Children’s Health, Beijing, China; 2 Department of Clinical Laboratory, Beijing Tsinghua Chang-gung Hospital, School of Clinical Medicine, Tsinghua University, Beijing, PR. China; 3 Key Laboratory for Medical Virology, National Institute for Viral Disease Control and Prevention, China CDC, National Health and Family Planning Commission. Beijing, PR. China; University of Hong Kong, HONG KONG

## Abstract

We retrospectively analyzed serum IgM antibodies (Abs) to influenza viruses from two tertiary hospitals in Beijing from December 2016 to February 2018. Samples from 36,792 patients, aged 0–98 years, were collected and tested. Among the patients, 923 children from two winter flu seasons were assayed with both antigens and IgM Abs to Flu A and Flu B and assigned as paired groups. Another 2,340 adults and 1,978 children with only antigen tested in the 2016 and 2017 winter flu seasons were named as unpaired groups. IgM Abs-positivity rates in children were 0.80% and 36.57% for Flu A and Flu B, respectively, peaking at 4–5 years of age. For adults, the Flu A and Flu B IgM Abs-positivity rates were 10.34% and 21.49%, respectively, peaking at 18–35 years of age. The trend of temporal distribution between the children and the adults was significantly correlated for IgM Abs to Flu B, but not for Flu A. Compared with unpaired groups, the detection rate of Flu A antigen was significantly higher than IgM Abs in children, whereas frequencies of IgM Abs were higher than antigen in adults. Incidence of Flu B antigen was sharply increased in 2017 winter than in the 2016 winter in both children and adults, but no concomitant increase was observed in IgM Abs to Flu B. For paired children groups, incidence of Flu B antigen in the 2017 flu season was significantly higher than that in the 2016 flu season; in contrast, positive rates of IgM Abs in the 2017 flu season were even lower than those in 2016. Considering antigen detection may reflect the Flu A/Flu B epidemic, our results indicate single-assayed IgM Abs were less effective in the diagnosis of acute influenza virus infection, and the use of this assay for epidemiology evaluations was not supported by these findings.

## Introduction

The epidemic of influenza is an important public health problem in worldwide [1]. Influenza virus genera A (Flu A) or B (Flu B) infections account for a vast majority of influenza cases in humans [2]. Most of these infections are asymptomatic or exhibit relatively mild symptoms, presenting as influenza-like illnesses (ILI). However, some patients with influenza exhibit severe symptoms, leading to hospitalization or even death, particularly in children, the elderly, and those with underlying chronic conditions [3]. Seasonal influenza results in approximately 250,000–500,000 deaths worldwide in each year [4].

Flu A has been isolated from various species, including humans, contributing to substantial viral heterogeneity, resulting in the production of various subtypes with the potential to cause human pandemic [5,6]. In contrast, Flu B evolved almost exclusively as a human pathogen, limiting the generation of new strains by reassortment [7,8]. Two major subtypes of Flu A and two lineages of Flu B viruses co-circulate and cause annual epidemics. In China, since the spread of swine-origin H1N1 in 2009 [9], most outbreaks of seasonal flu have been trigged by the transmission of pdmH1N1/2009 (pH1N1) and seasonal A (H3N2) (sH3N2), as well as the type B influenza virus Victoria lineage (B/Victoria) [10]. However, low immunization coverage and lack of type B influenza viruses of the Yamagata lineage (B/Yamagata) in the current trivalent influenza vaccine in China would promote its prevalence [11].

Indeed, during the 2017–2018 winter season, China experienced a national wide influenza epidemic. Reported influenza cases throughout the entire nation in winter of 2017 (December 2017 to February 2018) increased 5.7 times as compared with that in winter of 2016 (December 2016 to February 2017). According to unpublished data acquired from the Centers of Disease Control and Prevention (CDC) of China, at least half of the identified viruses belonged to the B/Yamagata lineage during the 2017 winter flu season [12,13]. The epidemics have highlighted the need for simpler and rapider assays to facilitate the diagnosis of influenza infections and differentiate the subtypes/lineages of the viruses. Reverse transcription-polymerase chain reaction (PCR)-based tests, influenza rapid diagnostic test (IRDT), and serological detection of specific antibodies (IgM Abs and IgG Abs) targeting the viruses were recommended to assist with influenza diagnosis from ILI cases in the clinical setting [14].

Developed since early 1980s, most publications about serum IgM Abs assay were based on complement-fixation tests [15], while the distribution and diagnostic features of IgM Abs in ILI cases are still unclear [16]. In this study, we retrospectively analyzed the IgM Abs to Flu A and Flu B in two tertiary hospitals in Beijing during the period from December 2016 to February 2018, covering 15 months and two winter flu seasons. Frequencies of IgM Abs to Flu A and Flu B were compared with the findings of contemporary antigen tests, providing insights into the use of IgM Abs for influenza diagnosis.

## Materials and methods

### Ethics statement

Data were analyzed anonymously. The studies did not involve any health-related patient information and were approved by the Ethics Committee of both Beijing Children’s Hospital and Beijing Tsinghua Changgung Hospital (approval no. 2018-k-130 / 17120-0-01).

### Case definition and study population

**Fig 1:**
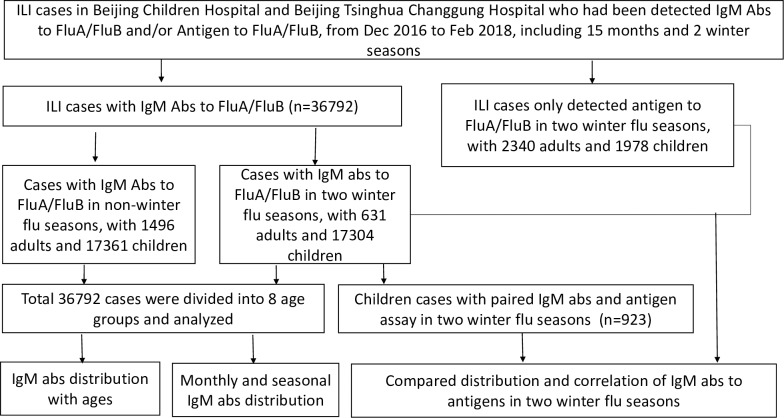
Roadmap indicating cases selection, subgroups, and data analysis procedures.

ILI cases were defined as previously described [17]. In total, 36,792 unrepeated patients with ILI who sought medical services at Beijing Children’s Hospital and Beijing Tsinghua Changgung Hospital from December 2016 to February 2018 were enrolled in this study. Patients ranged in age from 0 to 98 years old and were divided into eight age groups: 1,031 (2.80%) cases aged between 13 h and 30 days, 5,486 (14.91%) cases aged between 1 and 12 months, 11,950 (32.48%) cases aged 1 to 3 years, 5,608 (15.24%) cases aged 4 to 5 years, 10,590 (28.78%) cases aged 6 to 17 years, 402 (1.09%) cases aged 18 to 35 years, 529 (1.44%) cases aged 36 to 60 years, and 1,196 (3.25%) cases aged over 60 years. In the 36,792 cases, 519 children from the 2016 flu season (December 2016 to February 2017) and 404 children from the 2017 flu season (December 2017 to February 2018) were concomitantly assayed with direct immunofluorescence assay (DFA) for antigen to Flu A and Flu B and defined as paired groups. Other 2,340 adults and 1,978 children with Flu A and Flu B antigen tested by an IRDT method during 2016 and 2017 winter flu seasons were included as unpaired groups, with purpose to use the antigen as indicators for virus epidemics (Fig 1).

### Indirect immunofluorescence assay (IIFA) of IgM Abs

A Respiratory Tract Profile (IgM) kit (EUROIMMUN, Germany) was adopted by the two hospitals. Based on the titer plane technique using infected cells/cultured bacteria, this test kit is designed for the in vitro determination of human IgM Abs to Flu A, Flu B, and another six atypical respiratory pathogens, including adenovirus, respiratory syncytial virus, parainfluenza virus, chlamydia pneumoniae, legionella pneumophila, and mycoplasma pneumoniae using serum/plasma samples. Detections were performed qualitatively according to the manufacturer’s instruction: Apply 30 μl of diluted serum or plasma samples to each reaction field of the reagent tray, incubate for 30 mins at room temperature, then rinse the BIOCHIP slides with a flush of phosphate buffer solution (PBS) -Tween, followed by applying 25 μl of fluorescein labeled anti-human globulin to each reaction field, Incubate for 30 mins again, then wash with the new PBS-Tween for at least 5 mins, Place embedding medium onto a cover glass to read the fluorescence with the microscope. Fluorescence results were read by experienced technicians.

### DFA for detection of virus antigen

A D3 Ultra DFA Respiratory Virus Screening & ID Kit (DIAGNOSTIC HYBRIDS, USA) was used at Beijing Children’s Hospital for influenza antigen detection. This kit uses viral antigen-specific murine monoclonal antibodies that are directly labeled with fluorescein for the rapid detection and identification of seven respiratory viruses, including Flu A, Flu B, respiratory syncytial virus, adenovirus, parainfluenza 1, parainfluenza 2, and parainfluenza 3. Tests were performed manually, the sputum or alveolar lavage were collected, after addition of PBS and vortex, centrifuge to wash up mucus and set aside the supernatant for viral isolation. The prepared cell suspension were dripped onto a 10-well slide and air dried. Fix the cells to the slides using fresh, chilled acetone and incubated with DFA screening reagent. Rinse the stained cells using de-mineralized water, and then using a fluorescence microscope with magnifications between 200 to 400 times by experienced technicians.

### Influenza rapid diagnostic test (IRDT)

Clearview Exact Influenza A&B was used at Beijing Tsinghua Changgung Hospital for influenza antigen tests. This kit is a product of Alear subcompany (Abon Biopharm Co., Ltd., China). The reagent is coated with anti-influenza A and anti-influenza B NP in one strip, enabling differentiation between Flu A and Flu B. Undiluted swabs were used for the detection, and all specimens were tested in a single experiment. The IRDT method had been compared with RT-PCR methods, the positive consistency was 54.3% and negative consistency was 97.3% and the two methods had concordance at 86.9% for Flu B; the positive consistency was 47.83% and negative consistency was 98.65% and the two methods had concordance at 87.82% for Flu A [18].

### Statistical analysis

Statistical analysis was performed using SPSS (version 19.0; SPSS Inc., Chicago, IL, USA). For comparisons of categorical data, χ^2^ tests and Fisher’s exact tests were used as appropriate. To compare trends of monthly distributions of IgM Abs between children and adults, Spearman tests were performed for the correlation. All tests were two-tailed, and results with p values of 0.05 or less were considered statistically significant.

## Results

### Distributions of IgM Abs to Flu A and Flu B in different age groups

**Fig 2:**
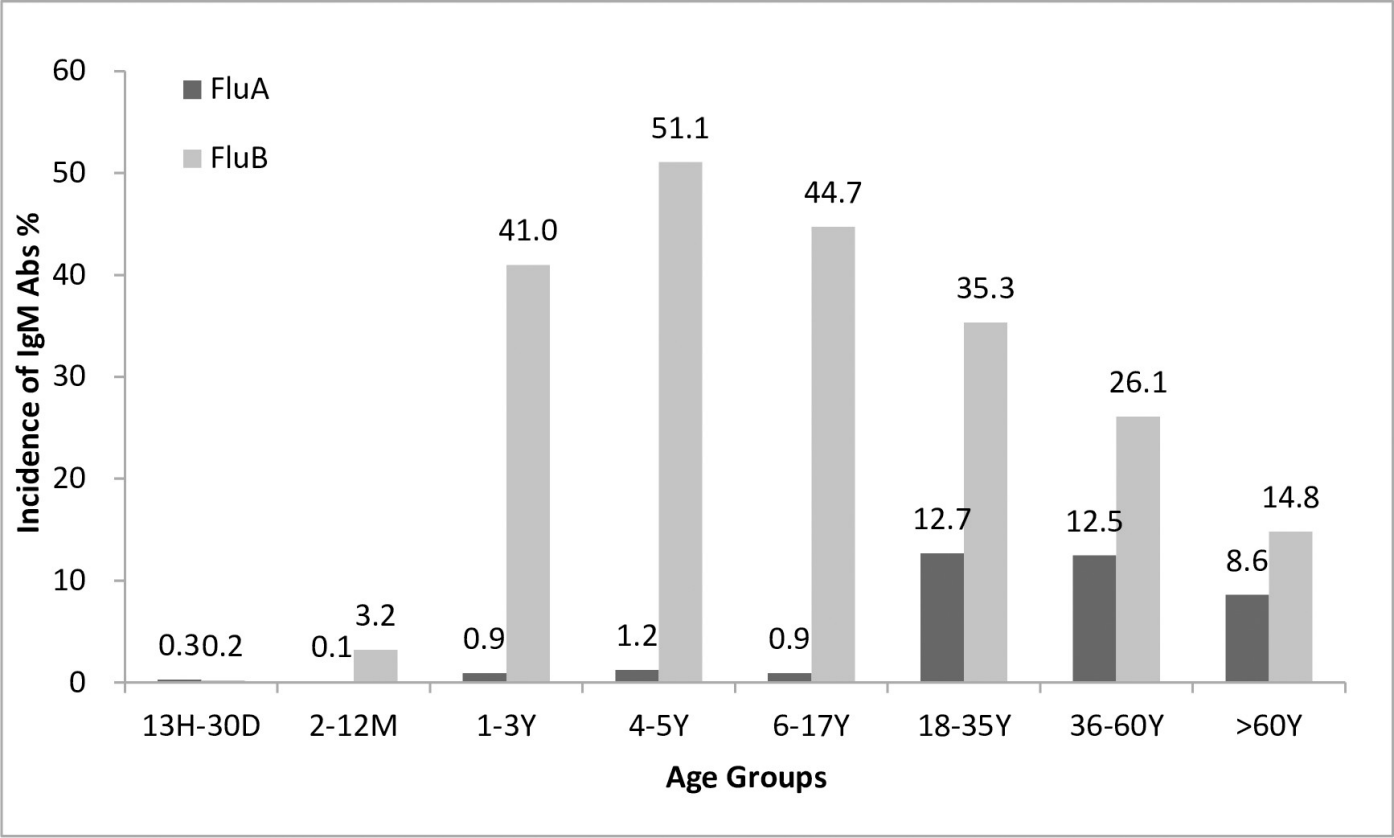
Distributions of IgM Abs for Flu A and Flu B in different age groups.

All 36,792 ILI cases were divided into eight age groups. Significant differences of the two IgM Abs distributions were found among groups (p < 0.01; Table 1). Generally, detection rates of IgM Abs were super low in patients less than 1 year of age. The detection of both IgM Abs peaked at 4–5 years of children, with frequencies of Flu A and Flu B at 1.21% and 51.07%, respectively (**Fig 2**). In pediatric cases, the general positivity rate of IgM Abs to Flu A was 0.80% (278/34665) and 36.57% (12676/34665) to Flu B. In adult cases, the general positivity rates of IgM Abs to Flu A and Flu B were 10.34% (220/2127) and 21.49% (457/2127) respectively.

**Table 1:** Distributions of IgM Abs to Flu A and Flu B in different age groups.

**Age group**	**IgM Abs**	**13 h–30 D**^b^ **(n = 1031)** **No. (%)**	**2–12 M**^b^ **(n = 5486)** **No. (%)**	**1–3 Y**^b^ **(n = 11950)** **No. (%)**	**4–5 Y**^b^ **(n = 5608)** **No. (%)**	**6–17 Y**^b^ **(n = 10590)** **No. (%)**	**18–35 Y**^b^ **(n = 402)** **No. (%)**	**36–60 Y**^b^ **(n = 529)** **No. (%)**	**> 60 Y**^b^ **(n = 1196)** **No. (%)**	***p* values**
**IgM Abs**	Total Positive^a^	4 (0.39)	179 (3.26)	4928 (41.24)	2877 (51.30)	4755 (44.90)	182 (45.27)	187 (35.34)	255 (21.32)	**<0.01**
	Co-positive^a^	1 (0.10)	1 (0.02)	75 (0.63)	55 (0.98)	79 (0.75)	11 (2.73)	17 (3.21)	25 (2.09)	**<0.01**
	Flu A	3 (0.29)	5 (0.09)	106 (0.89)	68 (1.21)	96 (0.91)	51 (12.69)	66 (12.47)	103 (8.61)	**<0.01**
	Flu B	2 (0.19)	175 (3.19)	4897 (40.98)	2864 (51.07)	4738 (44.74)	142 (35.32)	138 (26.08)	177 (14.79)	**<0.01**

The percentages were estimated as a fraction of total cases belonging to each category. *P* values were estimated using χ^2^ tests or Fisher’s exact tests (age groups), and *p* values of 0.05 or less are shown in bold.

^a^ Total positive: IgM Abs to Flu A or Flu B; Co-positive: IgM Abs to Flu A and Flu B.

^b^ h: hours; D: days; M: months; Y: years.

The detection rates of IgM Abs to Flu A were higher in adults than in children (p < 0.01), with the highest detection rate (12.69%) in the 18–35-year-old group (Table 1). The IgM Abs to Flu A declined to 8.61% in the over-60-year-old group, significance could be seen when compared to adult groups of 18–35 (P = 0.02) and 36–60 (p = 0.01) years old. Flu B IgM Abs were much more prevalent than Flu A IgM Abs in both children and adults, but its frequencies in children were much higher than in adults (p < 0.01), with the detection rate peaked in 4–5 year group, and then decreased gradually in other aged groups (p < 0.01) (**Fig 2**).

### The temporal distributions of Flu A and Flu B IgM Abs

**Fig 3:**
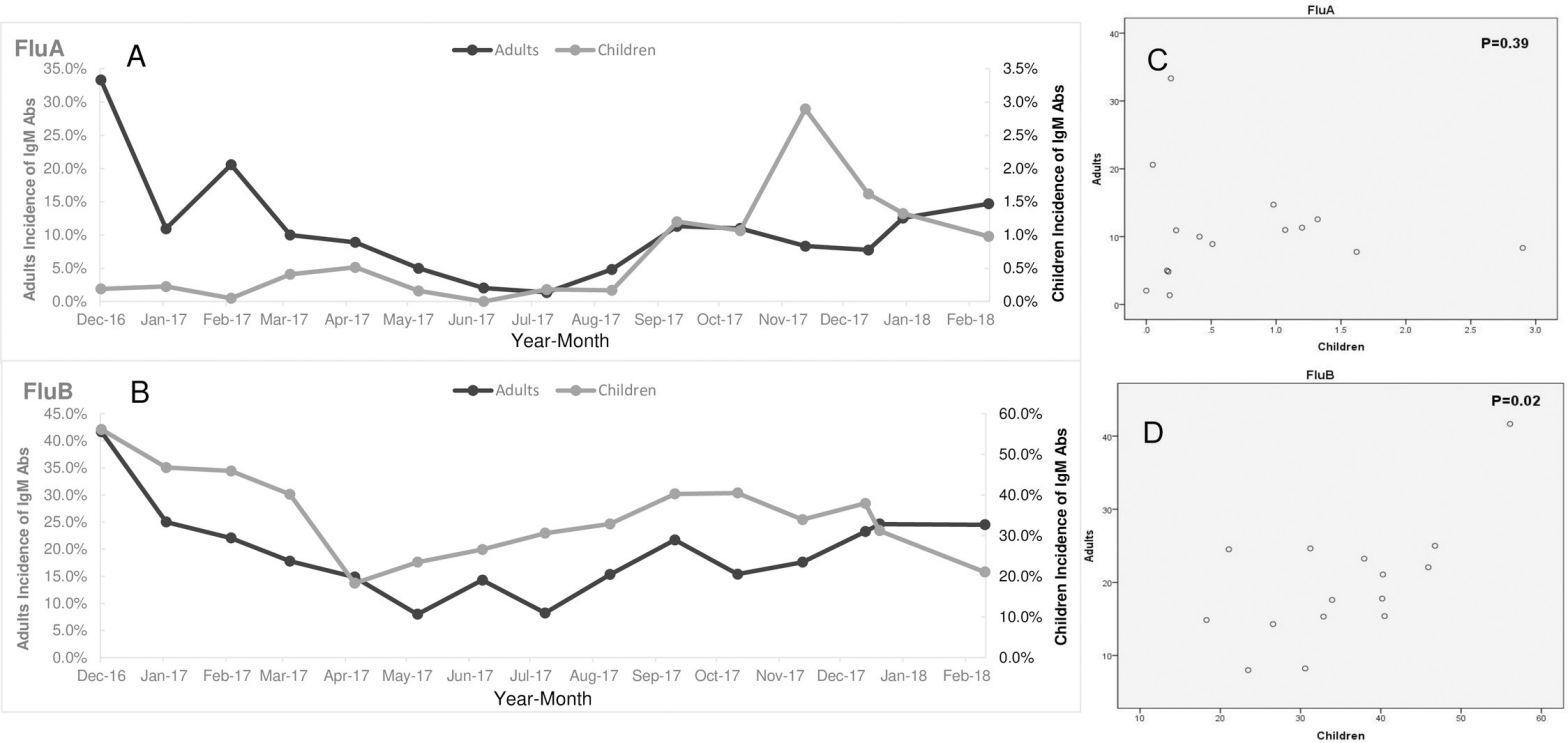
**Temporal distributions (from December 2016 to February 2018) of IgM Abs to Flu A and Flu B in pediatric and adult ILI cases.** A and B: monthly distributions of Flu A and Flu B IgM Abs. C and D: Comparison of monthly distributions of Flu A and Flu B IgM Abs between children and adults by spearman tests.

The data collected were covering from December 2016 to February 2018. The distributions of IgM Abs to Flu A and Flu B in children and adults were summarized in terms of temporal sequences. Generally, similar trends were observed between children and adults, with both IgM Abs increasing from autumn to winter and decreasing from spring to summer (Fig 3). For IgM Abs to Flu A, the monthly trends for children and adults were not correlated (p = 0.39), with two obvious discordances occurred. The first happened in December 2016, when there was a rapid increase in IgM Abs to Flu A in adults while a steady, low positivity rate in children. The second occurred from November 2017 to January 2018, when there was a sharp increase in IgM Abs to Flu A in children, but no changes in adults (Fig 3).

Significant correlation in the IgM Abs to Flu B could be found between children and adults (p = 0.02). Two gentle peaks presented, with the first occurring from December 2016 to February 2017, and the second occurring from September to November 2017. It is worth noting that in the winter of 2017, during the epidemic of the B/Yamagata lineage, the positive rate of IgM Abs to Flu B was consistently stable in adults and even decreased in children as compared with that in previous months (Fig 3).

### Comparing results of antigen and IgM Abs using the unpaired groups

**Fig 4:**
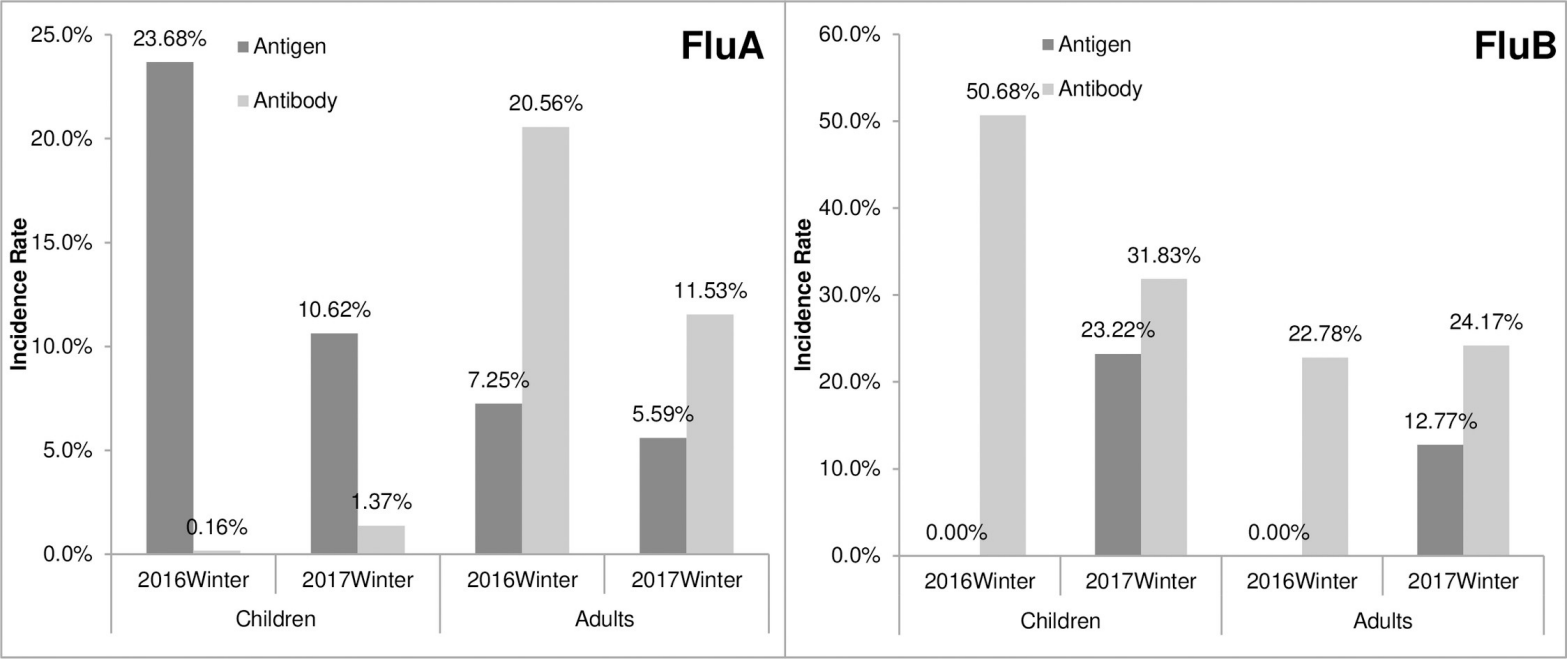
Antigen and IgM Abs distributions in unpaired pediatric and adult ILI cases in two winter flu seasons.

Considering that contemporaneous antigen results, which were detected at random, may indicate the epidemic intensities of certain pathogens, we compared the influenza antigen and IgM Abs results during the 2016 and 2017 winter flu seasons (Table 2, Fig 4). Results indicated that the positive rate of antigen and IgM Abs was not consistent for both Flu A and Flu B. In winter of 2016, the positivity rate of Flu A antigen in children was 23.68%, whereas IgM Abs to Flu A was 0.16% (p <0.01). In winter of 2017, the positivity rate of Flu A antigen in children was 10.62%, whereas that of IgM Abs to Flu A was 1.37% (p <0.01). Generally in children, the detection rate of Flu A antigen was significantly higher than the IgM Abs to Flu A in two consecutive winter flu seasons. The situation was conversed in adults. During the 2016 winter season, the positive rate of Flu A antigen was 7.25%, and that of IgM Abs to Flu A was 20.56% (p <0.01). During the 2017 winter season, the positivity rate of Flu A antigen was 5.59%, and that of IgM Abs to Flu A was 11.53% (p <0.01). The lower frequency of antigen and higher IgM Abs for Flu A in adults seems to indicate that adults are more inclined to develop IgM Abs for Flu A by an occult infection [19].

**Table 2:** Contemporary antigens and IgM Abs distributions in unpaired pediatric and adult ILI cases in two winters.

			**Antigen distribution**		**IgM Abs distribution**	
	Flu season	Month	Pos/Adults (N%)	Pos/Children (N%)	Pos/Adults (N%)	Pos/Children (N%)
**Influenza A**	2017	Feb-2018	22/574 (3.83%)	26/266 (9.77%)	15/102 (14.71%)	18/1833 (0.98%)
		Jan-2018	91/1463 (6.22%)	91/1055 (8.63%)	26/207 (12.56%)	45/3399 (1.32%)
		Dec-2017	14/234 (5.99%)	77/505 (15.24%)	11/142 (7.75%)	58/3583 (1.62%)
		total	127/2271 (5.59%)	194/1826 (10.62%)	52/451 (11.53%)	121/8815 (1.37%)
	2016	Feb-2017	1/14 (7.14%)	2/9 (22.22%)	14/68 (20.59%)	1/2076 (0.05%)
		Jan-2017	4/42 (9.52%)	26/106 (24.53%)	7/64 (10.94%)	6/2663 (0.23%)
		Dec-2016	0/13 (0)	8/37 (21.62%)	16/48 (33.33%)	7/3750 (0.19%)
		total	5/69 (7.25%)	36/152 (23.68%)	37/180 (20.56%)	14/8489 (0.16%)
**Influenza B**	2017	Feb-2018	61/574 (10.61%)	54/266 (20.30%)	25/102 (24.51)	386/1833 (21.06%)
		Jan-2018	197/1463 (13.47%)	255/1055 (24.17%)	51/207 (24.64)	1061/3399 (31.22%)
		Dec-2017	32/234 (13.68%)	115/505 (22.77%)	33/142 (23.24%)	1359/3583 (37.93%)
		total	290/2271 (12.77%)	424/1826 (23.22%)	109/451 (24.17%)	2806/8815 (31.83%)
	2016	Feb-2017	0/14 (0)	0/9 (0)	15/68 (22.06%)	953/2076 (45.91%)
		Jan-2017	0/42 (0)	0/106 (0)	16/64 (25.00%)	1245/2663 (46.75%)
		Dec-2016	0/13 (0)	0/37 (0)	20/48 (41.67%)	2104/3750 (56.11%)
		total	0/69 (0)	0/152 (0)	51/180 (28.33%)	4302/8489 (50.68%)

For Flu B detection, the positivity rate of Flu B antigen was zero in children, whereas the IgM Abs to Flu B was 50.68% (p <0.01) in the 2016 winter flu season. In the 2017 winter season, the positivity rate of Flu B antigen in children was 23.22%, but the IgM Abs to Flu B was 31.83% (p <0.01). In the 2016 winter season, the positivity rate of Flu B antigen in adults was zero, but the IgM Abs to Flu B was 28.33% (p <0.01). During the 2017 winter season, the positive rate of Flu B antigen was 12.77%, whereas the IgM Abs to Flu B was 24.17% (p <0.01). Antigen of Flu B was sharply increased during winter 2017 compared with that during the previous winter in both children and adults, and the result was consistent with the data released by the CDC of China[13]. However, no proportionate increase in IgM Abs to Flu B was found (Table 2, Fig 4).

### Comparison of antigen and IgM Abs distributions in paired pediatric groups

**Fig 5:**
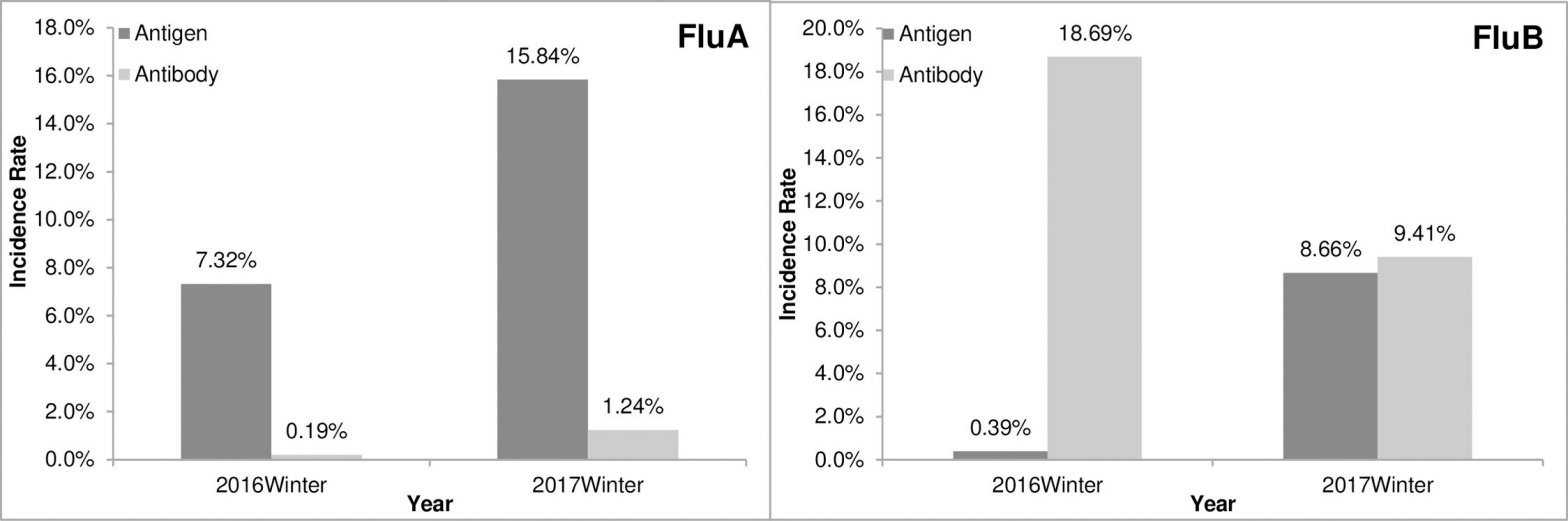
Comparison of results between IgM Abs and antigens in pediatric ILI cases in two winter flu seasons.

There were 923 hospitalized pediatric patients with ILI simultaneously analyzed for influenza antigen and IgM Abs (519 in the 2016 and 404 in the 2017 flu seasons). Significant differences between antigen and IgM Abs distribution were observed. The positivity rates of Flu A antigen were 7.32% in the 2016 winter season and 15.84% in the 2017 winter season (p <0.01), whereas in both seasons, the detection rate of IgM Abs to Flu A was less than 2%. The positivity rates of Flu B antigen were 0.39% in 2016 and 8.66% in 2017, whereas those of IgM Abs to Flu B were 18.69% in 2016 and 9.41% in 2017. Antigen detection of Flu B in the 2017 flu season was higher than the 2016 flu season (p <0.01); on the contrary, IgM Abs to Flu B in the 2017 flu season were even lower than those in the 2016 flu season (p <0.01; Fig 5). The inconsistent results between IgM Abs and antigen to Flu B were in line with results derived from the unpaired groups.

## Discussion

In clinical setting, ILI cases might be attributed to seasonal Flu A and Flu B virus infections, newly pandemic viruses, such as p(H1N1), and other respiratory pathogens [11,20]. Rapid and reliable pathogens screening is important for diagnosis and prevention of influenza virus infection. Common detecting methods include IRDT for Flu A and Flu B virus, single or multiplex nucleic acid assays for viral RNA, and serum-specific IgM or IgG Abs to influenza virus [21,22]. To date, many reports have described the development, evaluation, and application of the first two methods, but few reports have documented IgM Abs detection [15,16].

Detection of specific IgM Abs against viruses and other atypical pathogens has been widely applied for the diagnosis of respiratory infection [22]. Using enzyme-linked immunosorbent assays (ELISA) or profiled immunofluorescent assays, specific IgM Abs to Flu A and Flu B can be detected.

However, the assay is widely taken advantage for single detection use in the clinical setting. The improper use may lead to clinical misdiagnosis and mistreatment. A study once indicated that IgM Abs to Flu A, as determined by the IIFA method, could be used for diagnosis in 64% of single sera with complement-fixation Abs titred between 32 to 256 [23]. Another study indicated that using ELISA to detect IgM, IgG, and IgA Abs responses to influenza p(H1N1) hemagglutinin in infected persons during the 2009 pandemic exhibited lower sensitivity compared with hemagglutination inhibition and microneutralization assays. Cross-reactive Abs against homologous and heterologous subtypes did exist in serologic Abs studies [24], it is necessary to eliminate IgG Abs by immunoadsorption before the IgM Abs assay using profiled IIFA. Other Abs tests for H5N1 and H7N9 using ELISA also indicated limited sensitivity [25].

In this study, we reviewed and summarized the results of single serum IgM Abs to Flu A and Flu B viruses in 36,792 ILI cases, and compared IgM Abs with unpaired and paired antigens tests over the same period, with purpose to offer insights on rational screening methods selection facing the epidemic of Flu A and Flu B viruses. To the best of our knowledge, this is the first report demonstrating the correlation of IgM Abs to Flu A and Flu B with influenza antigen detection. Beijing Children’s Hospital and Beijing Tsinghua Changgung Hospital are two tertiary hospitals in Beijing; one is located at the south end of the city, and the other is located at the north end of the city. As a result, patients are representative of the epidemiology of the whole city. All IgM Abs had been screened using the IIFA product by EUROIMMUN at two hospitals.

The IgM Abs to Flu A and Flu B was related to ages. Notably, the rate of IgM Abs was very low in children less than 1 year old, whereas IgM Abs to Flu A and Flu B both increased significantly with aging. IgM Abs to Flu B peaked (51.07%) at 4–5 years and then declined gradually with age, while a considerably high frequency (14.79%) was maintained in over-60-years group. Children under 1 year age live in a relatively closed and safe environment and keep neutralizing antibodies from their mothers, thereby avoiding influenza virus infection. On other hand, children ages 4–5 years old attend kindergarten and usually they are active to public facilities and therefore have more exposure to influenza viruses [26]. The incidence of IgM Abs to Flu B in children was significantly higher than that to Flu A, consistent with reports demonstrating that children are more likely to acquire Flu B infection [10,27]. Studies have indicated that Flu B detection rates among ILI pediatric cases ranged from 9.5% to 56.4% during 2006 to 2017 in China [28,29]. This proportion was similar to those observed in the United States (0.1–44.6%) [30,31], but was higher than those in Europe (0–16.4%) [32]. So far, mismatches between the circulating strains and the type B strains in the trivalent vaccine were observed, suggesting higher clinical disease burden [27]. Oseltamivir, which is recommended the main antiviral drug for treatment of Flu A virus infection, has been shown to be less effective in shortening the duration of viral shedding and febrile illness in children with Flu B [29]. Such a high incidence of IgM Abs to Flu B indicates the severe disease burden and we should pay more attention to choosing screening methods.

IgM Abs to Flu A remained less than 2% in five pediatric groups and then reached to the peak in the 18–35-year-old group, subsequently showed a gradual decrease with age. The low positivity rate of IgM Abs to Flu A in children deserved further study to clarify. Our speculations to this result are, comparing with adults, children live in a relatively simple and secure environment, so the transmission route may be cut off; the immune response to Flu A virus is relatively week in children so less antibodies produced; some ILI symptoms in children are result of other respiratory virus infection. However, comparison of the frequency between antigen and IgM Abs may provide other insights. In both adults and children, antigen to Flu A was detected with a substantially higher rate in 2016 (23.68%) and 2017 (10.62%) winter flu seasons. This is in controversy to the considerably low rates of IgM Abs to Flu A in children (0.16% in 2016 and 1.37% in 2017). Of the 923 children with paired detection results of both antigen and IgM Abs, similar disagreement results were observed, antigen to Flu A was detected with 7.32% in 2016 and 15.84% in 2017 winter flu seasons, but the IgM Abs to Flu A were less than 2% in children. Flu weekly report from Chinese National Influenza Center had it that subtype H3N2 of Flu A was the major circulating strain in 2016 and 2017 winter flu seasons in China [12]. The antigen result indicates the severity of the Flu A epidemic was equivalent or more severe in children than adults. The disproportionately low IgM Abs to Flu A in children is worthy of attention. The results support the conclusion that currently used IgM Abs assays might miss detecting IgM Abs to Flu A in these children, or in the early stage of diseases.

Analysis of monthly distribution of IgM Abs indicated that both IgM Abs increased from autumn to winter and decreased from spring to summer. The trends of IgM Abs to Flu B were consistent between children and adults [33]. However, two waves of discordance for Flu A between children and adults were observed, one in December 2016 (increased in adults but smooth in children) and another in November of 2017 (increased in children but stable in adults). This could be explained by the erratic epidemic of Flu A especially with the subtype changing and diverse susceptibilities of adults and children to viruses. Studies had indicated that various subtypes of Flu A tend to infect people of different age. For example, p(H1N1) preferentially infects children under 14 years of age, whereas sH3N2 preferentially infects patients between 30 and 39 years of age, and sH1N1 infects patients more than 70 years of age [28].

There was an epidemic of the B/Yamagata strain in winter of 2017 in Beijing [12,13], the epidemic was also confirmed by our group using RT-PCR and by data from China CDC [13,18]. By analyzing unpaired and paired data of antigen to IgM Abs, we found that antigen to Flu B was significantly increased in 2017 winter compared with that in 2016, while there was no simultaneous increase in IgM Abs to Flu B. Since sensitivity of antigen detection by IRDT is lower than by RT-PCR [18,34], the actual Flu B infection should be severer than that indicated by antigen-positivity rate. Thus, some IgM Abs to Flu B in the 2017 winter flu season should have been present, but not detected by the single assay. Additionally, vaccinations [15,35] and immune memories in patients with sub-clinical infection [36] may also be contributed to higher IgM Abs detection. As a result, it is still unclear whether the positivity rate of IgM Abs to Flu B detected at a high frequency from 4–5 years old is a symbol of true infection or a reminiscent response. As China CDC administered a trivalent vaccine that does not include the B/Yamagata strain in 2017, we suspect that the IgM Abs to Flu B in 2017 winter might be true reflection of the Flu B virus infection.

There were some limitations of the present study. To determine the presence of influenza infection, the standard method should be virus culture combined with IIFA or hemagglutination inhibition tests in cultured cells. However, in this study, we compared IgM Abs screening data with antigen tests. We could not determine whether vaccination had an effect on IgM Abs detection. According to a survey, the general influenza vaccination rate in China is less than 3%, but approximately 10% in Beijing [27]. Therefore, the influence of vaccination on antibody detection could not be ignored. Also, the duration from getting infected to blood taking could not be confirmed, some negative results could be attribute to the window period of IgM Abs production.

In conclusion, we retrospectively reviewed the presence of IgM Abs to Flu A and Flu B in ILI cases from December 2016 to February 2018. The results indicated that IgM Abs were distributed across different age groups, with Flu A peaking in the 18–35-year-old group and Flu B peaking in the 4–5-year-old group. Seasonal distribution was also observed. Of clinical significance, single assayed IgM Abs were less effective for the diagnosis of acute influenza virus infection owing to lack of detection of IgM Abs to Flu A in two winter flu seasons in children and IgM Abs to Flu B in the 2017 winter flu epidemic in both children and adults. These results do not support the use of single IgM Abs assay for diagnosis and epidemiological evaluations. Thus, further studies are needed to develop new broad spectrum of influenza diagnostic assays based on serum.

## Supporting information

S1 Table P values of comparing the positive detection rate of IgM Abs between age groups for Flu A and Flu B.
(TIF)
