# Supplementary figures and images for: Unreliable usage of a single influenza virus IgM antibody assay in influenza-like illness: A retrospective study of the 2016–2018 flu epidemic
Source: PLoS One. 2019 Apr 22;14(4):e0215514. doi: 10.1371/journal.pone.0215514 (PMC6476501; doi:10.1371/journal.pone.0215514)

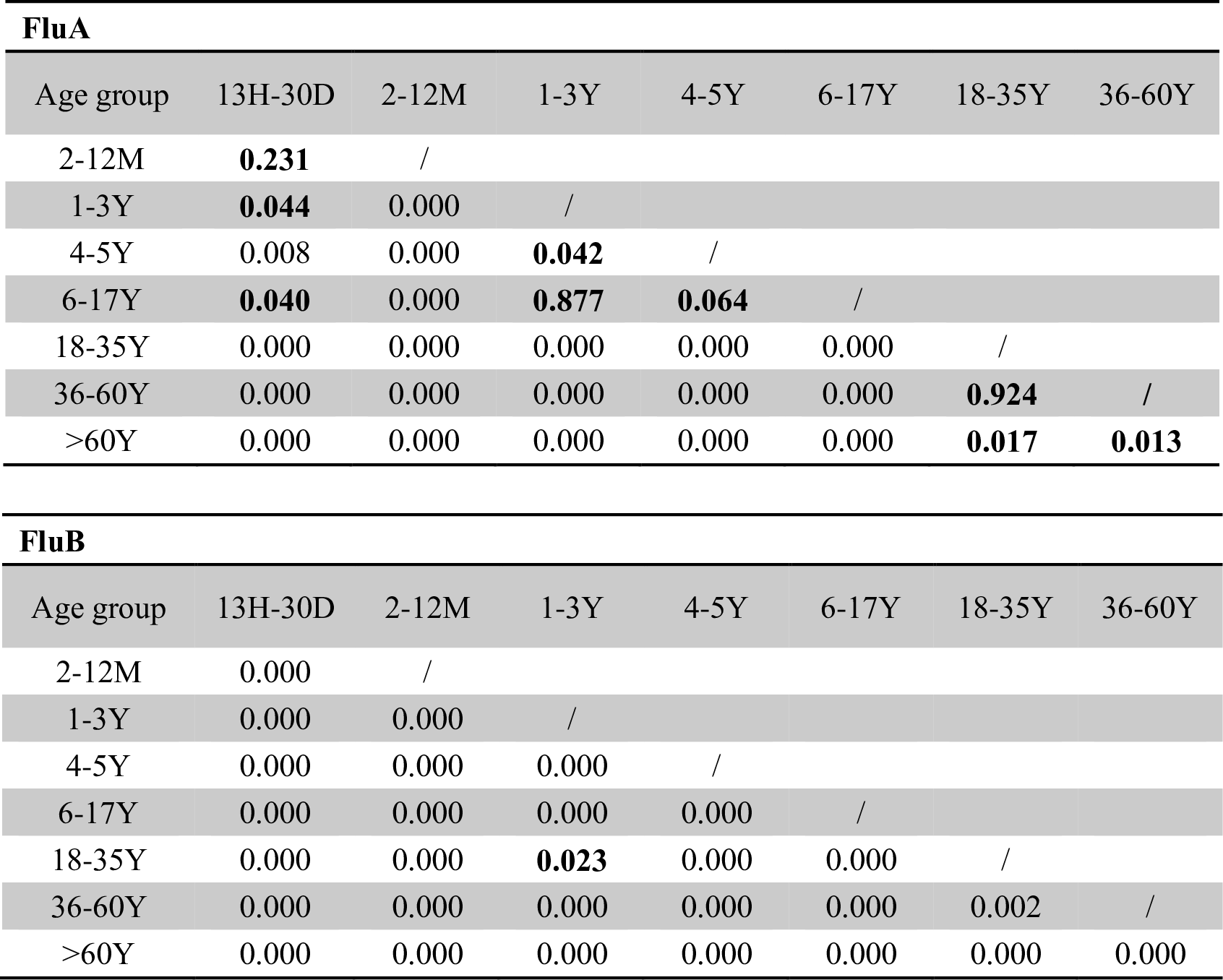

Supplement: S1 Table — P values of comparing the positive detection rate of IgM Abs between age groups for Flu A and Flu B. (TIF) [file pone.0215514.s001.tif]
